# Cystatin-like protein of sweet orange (C*sin*CPI-2) modulates pre-osteoblast differentiation via β-Catenin involvement

**DOI:** 10.1007/s10856-021-06504-y

**Published:** 2021-03-22

**Authors:** Célio da Costa Fernandes, Victor Manuel Ochoa Rodríguez, Andrea Soares-Costa, Joni Augusto Cirelli, Daniela Morilha Neo Justino, Bárbara Roma, Willian Fernando Zambuzzi, Gisele Faria

**Affiliations:** 1 Department of Chemistry and Biochemistry, Laboratory of Bioassays and Cell Dynamics, Institute of Biosciences, Sao Paulo State University – UNESP, Botucatu, São Paulo Brazil; 2 Department of Restorative Dentistry, School of Dentistry at Araraquara, Sao Paulo State University – UNESP, Araraquara, São Paulo Brazil; 3 Department of Genetic and Evolution, Federal University of Sao Carlos, São Carlos, Brazil; 4 Department of Diagnosis and Surgery, School of Dentistry at Araraquara, Sao Paulo State University–UNESP, Araraquara, São Paulo Brazil

## Abstract

Phytocystatins are endogenous cysteine-protease inhibitors present in plants. They are involved in initial germination rates and in plant defense mechanisms against phytopathogens. Recently, a new phytocystatin derived from sweet orange, C*sin*CPI-2, has been shown to inhibit the enzymatic activity of human cathepsins, presenting anti-inflammatory potential and pro-osteogenic effect in human dental pulp cells. The osteogenic potential of the C*sin*CPI-2 protein represents a new insight into plants cysteine proteases inhibitors and this effect needs to be better addressed. The aim of this study was to investigate the performance of pre-osteoblasts in response to C*sin*CPI-2, mainly focusing on cell adhesion, proliferation and differentiation mechanisms. Together our data show that in the first hours of treatment, protein in C*sin*CPI-2 promotes an increase in the expression of adhesion markers, which decrease after 24 h, leading to the activation of Kinase-dependent cyclines (CDKs) modulating the transition from G1 to S phases cell cycle. In addition, we saw that the increase in ERK may be associated with activation of the differentiation profile, also observed with an increase in the B-Catenin pathway and an increase in the expression of Runx2 in the group that received the treatment with C*sin*CPI-2.

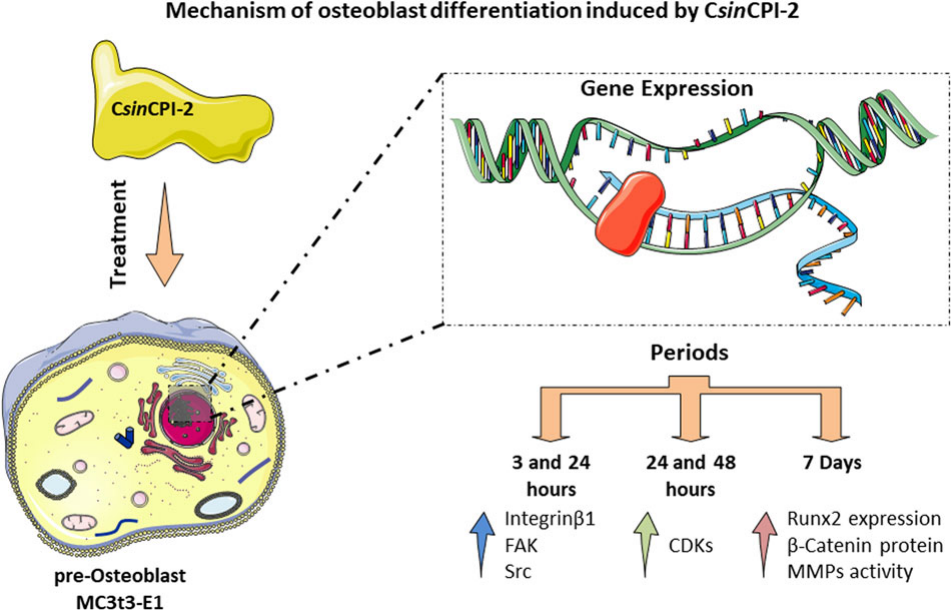

## Introduction

Bone constantly being in remodeling in a dynamic process requiring coupling of specialized cells and molecules [1], depends on the activity of proteases for breaking-down organic matrix components, such as matrix metaloproteinases (MMP) and cysteine cathepsins, mainly cathepsin K [2–4].

In turn, the cathepsins degrade the proteins of the extracellular matrix as collagen, laminin, fibronectin and proteoglycans [3] and participate of physiological processes, as tissue remodeling, turnover of the extracellular matrix, inflammation signaling, among others [5–7]. On the other hand, these enzymes also participate in diseases involving tissue remodeling as osteoporosis, osteoarthritis, cancer, cardiovascular diseases, apical periodontitis and other inflammatory diseases [3, 6–8]. Actually, there are 11 cathepsins encoded in the human genome – B, H, L, S, C, K, O, F, V, X e W [9]; among them, cathepsin K has a key role in bone resorption and is closely related to bone diseases as osteoporosis. For this reason, there is growth pharmaceutical interest focuse on developing the cathepsin K inhibitors to control the bone resorption, although cathepsin K inhibitors have failed in osteoporosis clinical trials [7].

In mammals, the major regulators of the activity of cysteine cathepsins are their own endogenous inhibitors named cystatins [3]. The endogenous cysteine- protease inhibitors are also present in plants and they are named phytocystatins [10]. Phytocystatins participate in the regulation of cysteine proteases during programmed cell death, leaf senescence [11] and have been related on control of phytopathogens [11, 12]. Phytocystatins has also been studied in crop genetic improvement [11]. Moreover, recombinants phytocystatins have shown potential to be used in different therapeutic approaches. Cystatins derived from sugarcane reduce dental enamel erosion [13], inhibit the causative agent of malaria [14] and the development of melanoma in vivo [15]. The cystatin derived from rice bran can be used in the production of health-promoting bioactive peptides for functional food formulation [16].

Recently, a new phytocystatin derived from *Citrus sinensis* (sweet orange), C*sin*CPI-2, was identified and recombinantly produced [17, 18]. The C*sin*CPI-2 has shown inhibiting the human cathepsins K [17, 18] and B [18], and presenting important anti-inflammatory potential and also pro-osteogenic effect. This pro-osteogenic effect was demonstrated in culture of human dental pulp cell, which is a physiological source of stem cells. When those cells were exposed to C*sin*CPI-2, they showed higher alkaline phosphatase activity, mineralized nodule production and expression gene of the osteogenic markers, when compared to untreated cells [18]. In sum, the osteogenic potential of the C*sin*CPI-2 represents a new insight into cysteine proteases inhibitors of plants and this effect needs to be better addressed. Thus, the aim of this study was to investigate the performance of pre-osteoblasts in response to C*sin*CPI-2, mainly focusing on cell adhesion, proliferation, and differentiation mechanisms.

## Materials and methods

### Biological macromolecule and antibodies

The following antibodies were purchased from Cell Signaling (Danvers, MA) as follows: Cofilin (#3318, 19 kDa); Phospho-Cofilin (Ser3) (#3311, 19 kDa); MAPK Erk1/2 (#4695, 44/42), Phospho-Erk (Thr202/Tyr204) (#8544, 44/42); β-Catenin (#9582, 92 kDa); BMP7 (#4693, 49 kDa); GAPDH (#2118, 37 kDa). The production of C*sin*CPI-2 recombinant protein was performed in *E.coli* system and purification was performed through affinity chromatography in a nickel column, as described previously [18].

### Cell culture and acquisition of osteogenic phenotype

Mouse pre-osteoblasts, MC3T3-E1 - subclone 4 (ATCC CRL-2593), were maintained in αMEM medium supplemented with 10% Fetal Bovine Serum (FBS) at 37 °C and 5% CO2 [19]. To induce osteogenic differentiation, we treated pre-osteoblasts (MC3T3-E1, subclone 4) with ß-Glycerophosphate (10 mM) + Dexamethasone (0.03 g/mL) + Ascorbic Acid (50 μg/mL) (Sigma-Aldrich) culture with 10% FBS [20, 21].

### Cell viability assay

MC3T3 pre-osteoblastic cells were seeded in 96-well plates at a density of 5 x 10^4^ cells mL^−1^. After 24 h of seeding, the medium was removed, C*sin*CPI-2 was added into the cultures different concentrations up to 24 h, when the treating medium was removed and FBS-free containing MTT salt (1 mg/ml) was added and the cells were kept in an incubator up to 3 h. The incorporation of the MTT salt was evaluated by absorbance 570 nm, using SYNERGY-HTX multi-mode reader plate (Biotek, USA).

### Cell adhesion assay

For analysis of incorporation of violet crystal, the cells were trypsinized, resuspended and plated (96-well) with control medium or in medium containing C*sin*CPI-2 in density 50 x 10^4^ cells mL^−1^. After 24 h in culture, the medium was removed and the cell adhesion estimated by incorporating the violet crystal profile, measured at 540 nm using SYNERGY-HTX multi-mode reader (Biotek, USA).

### Wound healing

The wound-healing assay allows evaluating cell migration and possible cell interactions. To this end, pre-osteoblast were grown on coverslips and after the confluence stage, an injury was made in the middle of the cultures using a 1 mL sterile tip. After 3 and 16 h, these coverslips containing the cells were fixed for 1 h in 4% paraformaldehyde and stained with toluidine blue for 0.5 min on a hot plate (previously heated to 95 °C). Afterward, they were technically processed to acquire images using a conventional inverted microscope Axio Vert.A1 (Zeiss, Germany)

### Analysis of gene expression was performed by qPCR technology

Following the experimental design, after the treatment periods and respecting the cell adhesion (3 and 24 h), proliferation (24 and 48 h) and differentiation (7 days) phenotypes. The cells were collected in TriZOL for total RNA extraction. Then, the cDNA was synthesized using the High-Capacity cDNA Reverse Transcription Kit (Applied Bio-systems, Foster City, CA). For expression analysis, 10 µl final (5 µl Sybr, 0.5 µl Forward and 0.5 µl Reverse, 3 µl of water and 1 µl of sample) were used for gene expression. The evaluated genes were as follow: Cofilin, Integrin β1, Src, FAK, BMP-2, RUNX2, Osterix, ALP, BSP, OTC, OTP, CDK2, CDK4, p21, p15 and GAPDH (Table 1).

**Table 1:** Expression primer sequences and PCR cycle conditions

Gene	Primer	5’-3’ Sequence	Reaction’s Condition
Integrin β1	Forward	CTGATTGGCTGGAGGAATGT	95 °C-3 s; 55 °C-8 s; 72 °C-20 s
	Reverse	TGAGCAATTGAAGGATAATCATAG	
Src	Forward	TCGTGAGGGAGAGTGAGAC	95 °C-3 s; 55 °C-8 s; 72 °C-20 s
	Reverse	GCGGGAGGTGATGTAGAAAC	
FAK	Forward	TCCACCAAAGAAACCACCTC	95 °C-3 s; 55 °C-8 s; 72 °C-20 s
	Reverse	ACGGCTTGACACCCTCATT	
Cofilin	Forward	CAGACAAGGACTGCCGCTAT	95 °C-3 s; 55 °C-8 s; 72 °C-20 s
	Reverse	TTGCTCTTGAGGGGTGCATT	
BMP2	Forward	GGTCACAGATAAGGCCATTGC	95 °C-3 s; 55 °C-8 s; 72 °C-20 s
	Reverse	GCTTCCGCTGTTTGTGTTTG	
Runx2	Forward	GGACGAGGCAAGAGTTTCA	95 °C-3 s; 55 °C-8 s; 72 °C-20 s
	Reverse	TGGTGCAGAGTTCAGGGAG	
Osterix	Forward	CCCTTCCCTCACTCATTTCC	95 °C-3 s; 55 °C-8 s; 72 °C-20 s
	Reverse	CAACCGCCTTGGGCTTAT	
ALP	Forward	GAAGTCCGTGGGCATCGT	95 °C-3 s; 55 °C-8 s; 72 °C-20 s
	Reverse	CAGTGCGGTTCCAGACATAG	
BSP	Forward	GTACCGGCCACGCTACTTTCT	95 °C-3 s; 55 °C-8 s; 72 °C-20 s
	Reverse	GTTGACCGCCAGCTCGTTTT	
CDK2	Forward	TACCCAGTACTGCCATCCGA	95 °C-3 s; 55 °C-8 s; 72 °C-20 s
	Reverse	CGGGTCACCATTTCAGCAAA	
CDK4	Forward	TCGATATGAACCCGTGGCTG	95 °C-3 s; 55 °C-8 s; 72 °C-20 s
	Reverse	TTCTCACTCTGCGTCGCTTT	
p21	Forward	CGCCGATCAGCAGTATGAGT	95 °C-3 s; 55 °C-8 s; 72 °C-20 s
	Reverse	GCCGGGCTCTGGAACTTTAT	
p15	Forward	GGGCAAGTGGAGACGGTG	95 °C-3 s; 55 °C-8 s; 72 °C-20 s
	Reverse	ACCCCCGCTACCTGGATT	
GAPDH	Forward	AGGCCGGTGCTGAGTATGTC	95 °C-3 s; 55 °C-8 s; 72 °C-20 s
	Reverse	TGCCTGCTTCACCACCTTCT	

### Western blotting analyze

The cells were harvested into lysis buffer, containing: Tris [hydroxymethyl) aminomethane] - 50 mM] -HCl [pH 7.4], 1% Tween 20, 0.25% of sodium deoxycholate, 150 mM NaCl, 1 mM EGTA (ethylene glycol tetraacetic acid), 1 mM O-vanadate, 1 mM NaF and protease inhibitors [1 μg/ml aprotinin, 10 μg/ml leupeptin and 1 mM 4-(2-amino-ethyl)-benzolsulfonyl-fluoride-hydrochloride]. The pool of protein extracts was later resolved into SDS-PAGE (10%) and thereafter transferred into PVDF membrane (Bio-Rad, Hercules, CA, USA). Immediately, the membranes were blocked with 1% fat-free dry milk or bovine serum albumin (2.5%) in Tris-buffered saline (TBS) - between 20 (0.05%) and incubated overnight at 4 °C with appropriate primary antibody at 1:1000 dilution overnight. After washing in TBS-Tween 20 (0.05%), the membranes were incubated with secondary anti-rabbit, anti-goat or anti-rat IgGs conjugated to horseradish peroxidase, in dilutions of 1:5,000, in buffer of block for 1h at room temperature. Later, Enhance Chemiluminescence (ECL, Pierce, USA) was used to detect the bands.

### Staining with Alizarin RedS and alkaline phosphatase (ALP)

The pre-osteoblasts were plated (5 x 10^4^ cells mL^−1^) in 24-well plates and were treated in semi-confluence with C*sin*CPI2 for up to 7 days. The medium was changed every 3 days. ALP staining was performed as recommended by the manufacturer (SIGMAFAST BCIP/NBT tablet). For Alizarin RedS staining, the cells were fixed with 4% formalin for 45 min at room temperature, then 1% dye was added to the cultures and the plate was held in a darkroom for 45 min. Finally, the wells were washed with PBS and the plate was photographed using an inverted microscope (Zeiss, Germany).

### Picrossirius staining

For qualitative analysis of the collagen content, the cells were washed in phosphate buffered saline (PBS) and fixed in 4% paraformaldehyde for 1 h and then stained for 90 min in a 0.1% solution of red Sirius in picric acid aqueous, saturated, pH 2. Cells were then washed for 2 min in 0.01 n HCl and counterstained with Hematoxylin Harris for 6 min, washed in 70% ethanol, dehydrated and mounted using Permount. Finally, the wells were washed with PBS and the plate was photographed using an inverted microscope (Zeiss, Germany).

### Zymogran

Considering the experimental desing, the medium was collected and centrifuged to avoid cell debris, and the protein concentration determined by the Lowry method [22]. The gelatinolytic activities of the samples was evaluated by the fractionating of metalloproteinases (MMP-2 and MMP-9) into 12% polyacrylamide gel containing 4% gelatin, when the MMPs were renatured in the Triton X-100 aqueous solution (2% w/v), incubated for 18 h in renaturating buffer (Tris-CaCl 2) at 37 °C and after stained with Coomassie Blue R-250 0.05% for 3 h [23].

### Statistical analysis

The results were plotted as mean ± standard deviation (SD). One-way ANOVA followed by Tukey multiple comparisons test or t test were performed using GraphPad Prism version 6.0 for Mac OS, (GraphPad Software, San Diego, California USA, www.graphpad.com).

## Results

**Fig. 1:**
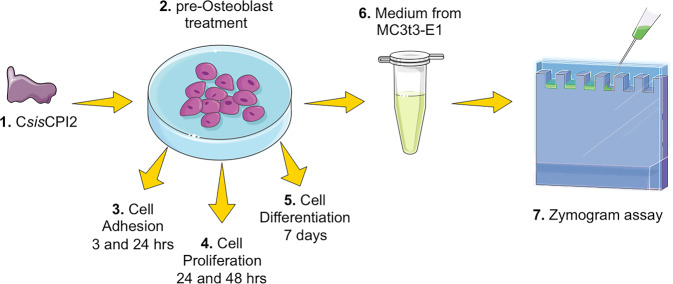
Experimental design. The isolated protein C*sin*CPI-2 (1) was used to treat pre-Osteoblasts (2) that were evaluated when adhering to the processes (3), proliferation (4) and differentiation (5) that at the end of the treatment had the medium collected (6) for investigation of MMPs by Zymography (7)

To better understand the possible involvement of C*sin*CPI-2 in the performance of osteoblasts, we investigated different signaling pathways after determining the cytotoxic effect (Fig. 1).

### Modulation of cell proliferation in pre-osteoblasts responding to C*sin*CPI-2

**Fig. 2:**
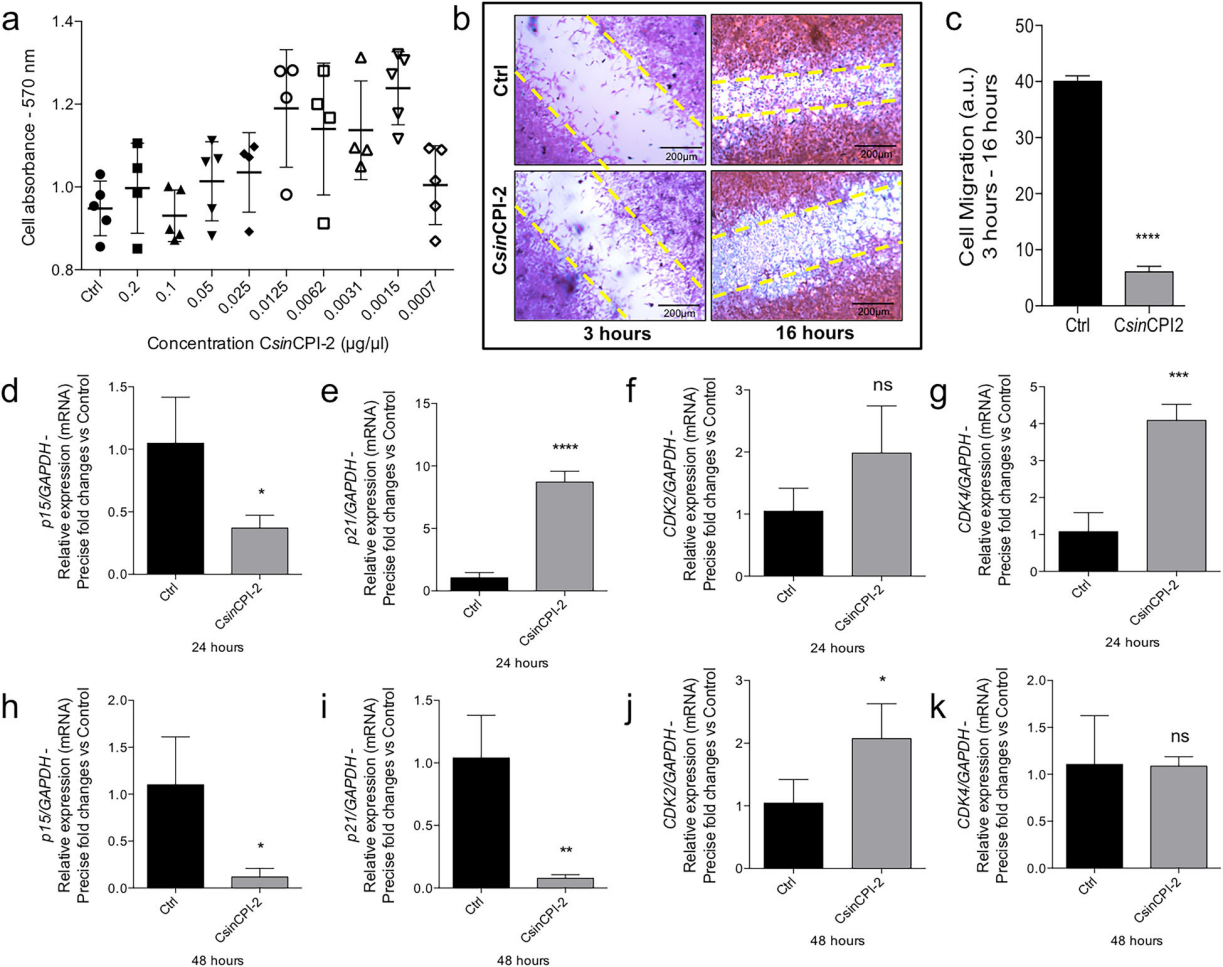
Cell proliferation and migration in response to C*sin*CPI-2. Pre-osteoblasts were evaluated for the cytotoxicity of the C*sin*CPI-2 protein in different concentrations (**a**) by MTT assay. The cell migration process was evaluated by a staining test after 3 and 16 h of aggression to the cell mat (**b**, **c**). To assess the proliferation process, cells were collected in Trizol and genes involved in the progression of the cell cycle investigated by real-time PCR: p15, p21, CDK2 and CDK4 (**d**–**k**). GADPH was considered housekeeping gene, and used to normalizing the gene expression. Statistical difference when compared to the control group: *p < 0.05; **p < 0.0082; ***p < 0.0002 and ****p < 0.0001

**Fig. 3:**
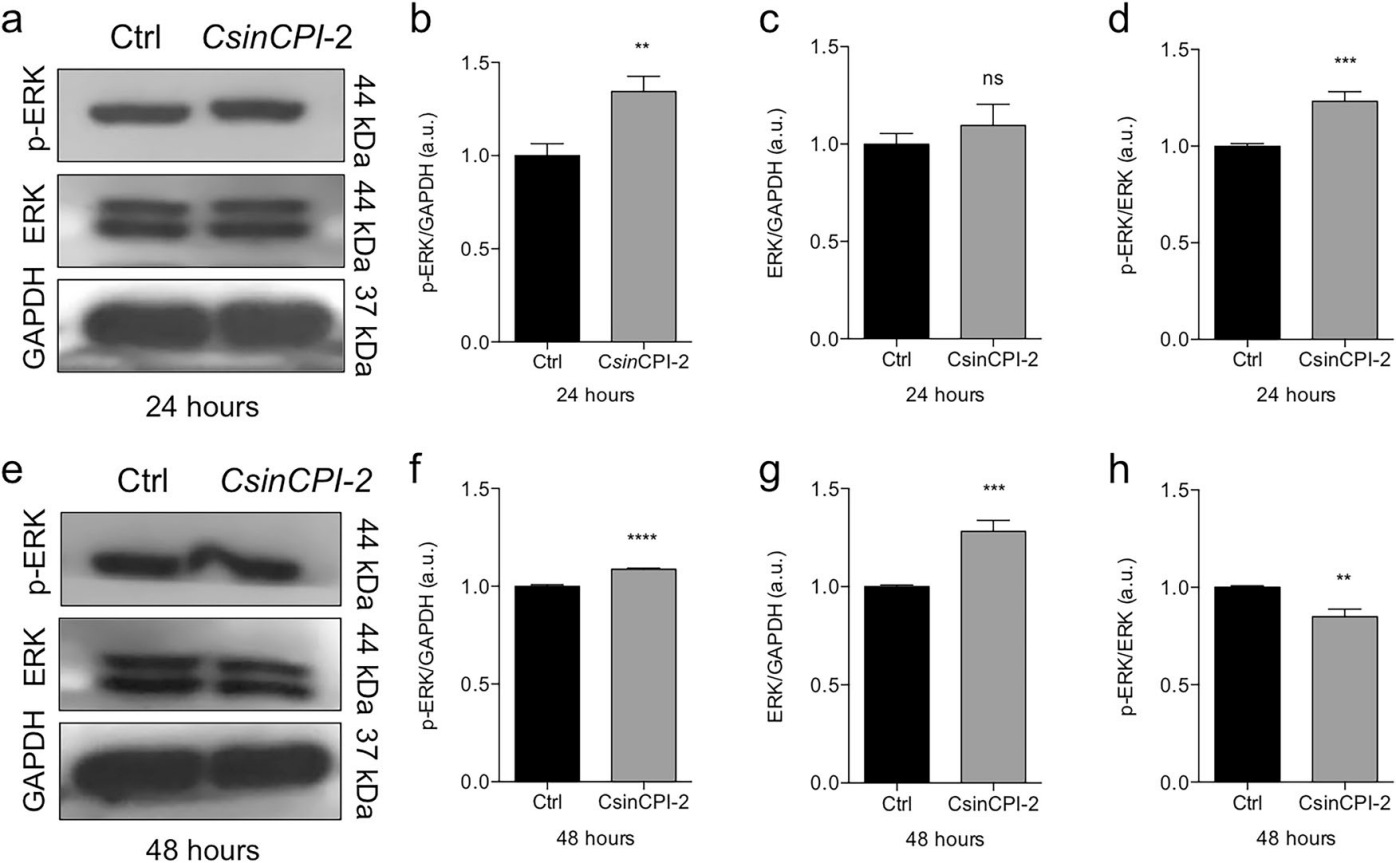
MAPK-ERK phosphorylation profile. To evaluate the ERK phosphorylation profile, cells were lysed after 24 and 48 h and the protein content revealed by SDS-PAGE 10%. ERK, p-ERK (Thr202/Tyr204). GAPDH were used as reference gene (**a**–**h**). GADPH was considered housekeeping gene, and used to normalizing the gene expression, as well as the protein expression by western blotting. Statistical difference when compared to the control group: **p < 0.0082; ***p < 0.0002 and ****p < 0.0001

Initially, we investigated the possible cytotoxicity profile of the C*sin*CPI-2 protein in pre-osteoblasts. Our results show that there is no significant effect on cell viability at the different concentrations investigated in this study (Fig. 2a). Although there was no statistical difference in the different doses, we observed a tendency to increase viability in the 0.0125 µg/µl dose, which led us to hypothesize whether there could be an increase in the proliferative process. To assess our hypothesis, we initially investigated the cell migration profile w of treatment. Comparing with the control group, at 16 h of treatment, the C*sin*CPI-2 protein does not promote an increase in the migration process (Fig. 2b, c). Another technology used to assess the progression of the cell cycle was qPCR; here we investigate the expression of genes that control the transition of the cell cycle phases. We observed that after 24 or 48 h of treatment, *p*15 was decreased in the treated group (Fig. 2d, h), while *p*21 and CDK4 cyclin was increased in 24 h (Fig. 2e, g). Finally, we investigated the phosphorylation profile of MAPK-ERK, which may indicate, additionally to other processes, the activation of the proliferation and survival mechanisms. The data reveal that after 24 h there is an increase in the content of phospho-ERK, whereas in 48 h this ratio presents an opposite effect (Fig. 3a–h).

### Behavior of pre-osteoblasts during adhesion when submitted to C*sin*CPI-2 protein

**Fig. 4:**
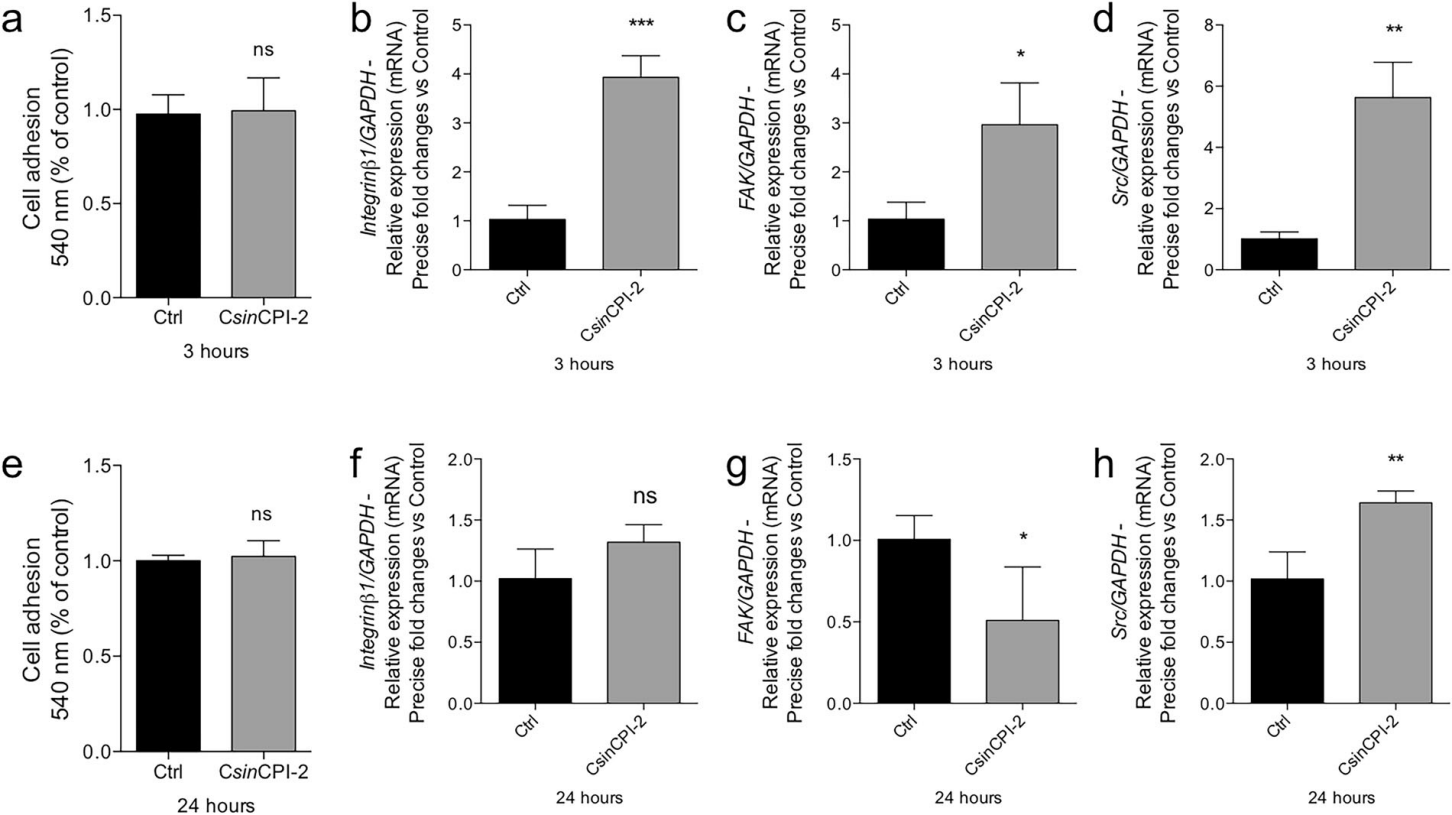
*Csin*CPI-2 protein modulates the pre-osteoblast adhesion profile. After 3 and 24 h of treatment, the cells were evaluated for cell adhesion profile by violet crystal assay (**a**, **e**). The cells were collected in TriZOL for RNA extraction, cDNA synthesis and investigation of the gene expression, as follows: Integrin (**b**, **f**), FAK (**c**, **g**) and Src (**d**, **h**) by real-time PCR. GADPH was considered housekeeping gene. Statistical difference when compared to the control group: *p < 0.05; **p < 0.0082 and ***p < 0.002

For the violet crystal colorimetric assay, there was no statistically significant difference between the control group and the group that received the C*sin*CPI-2 protein (Fig. 4a, e), but results of gene expression shows that the treatment positively modulated the expression of genes involved in the adhesion process. There was an increase in Integrin, Focal Adhesion Kinase (FAK) and Src over 3 h (Fig. 4b–d), while in 24 h there was a decrease in FAK expression, and an increase in Src gene (Fig. 4g, h).

**Fig. 5:**
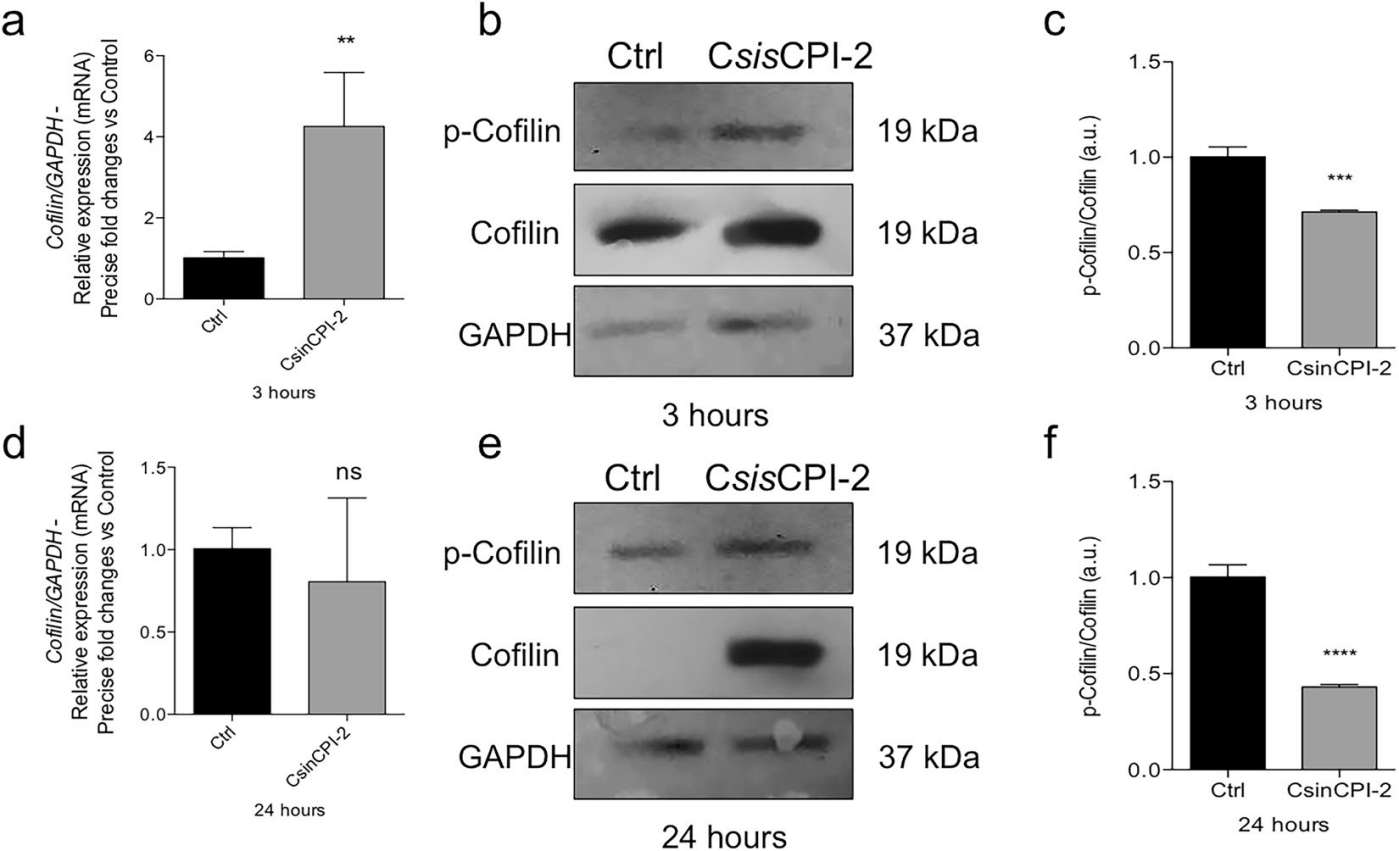
Cytoskeleton rearrangement of osteoblasts responding to C*sin*CPI-2. MC3T3 pre-osteoblast cells were harvested in TriZOL for total mRNA collection. The material was used for cDNA synthesis and investigation of cofilin expression (**a**, **d**). The cells were collected, lysed and the contents of cofilin and p-cofilin revealed by SDS-PAGE in 3 (**b**, **c**) and 24 h (**e**, **f**). GADPH was considered housekeeping gene, and used to normalizing the gene expression, as well as the protein expression by western blotting. Statistical difference when compared to the control group: **p < 0.0082; ***p < 0.0002 and ****p < 0.0001

It is very known that cell adhesion requires intense and dynamic cytoskeleton rearrangement, mainly coordinated by Cofilin driving actin filaments distribution. The data shows that after 3 h of treatment with C*sin*CPI2, there was an increase in the expression of Cofilin, whereas the protein ratio of p-Cofilin/Cofilin was decreased in the treated group (Fig. 5a–c). However, levels of expression do not change and the protein profile remained with a reduced ratio in 24 h (Fig. 5d–f).

### Osteogenic biomarker genes and ECM remodeling in response to C*sin*CPI-2

**Fig. 6:**
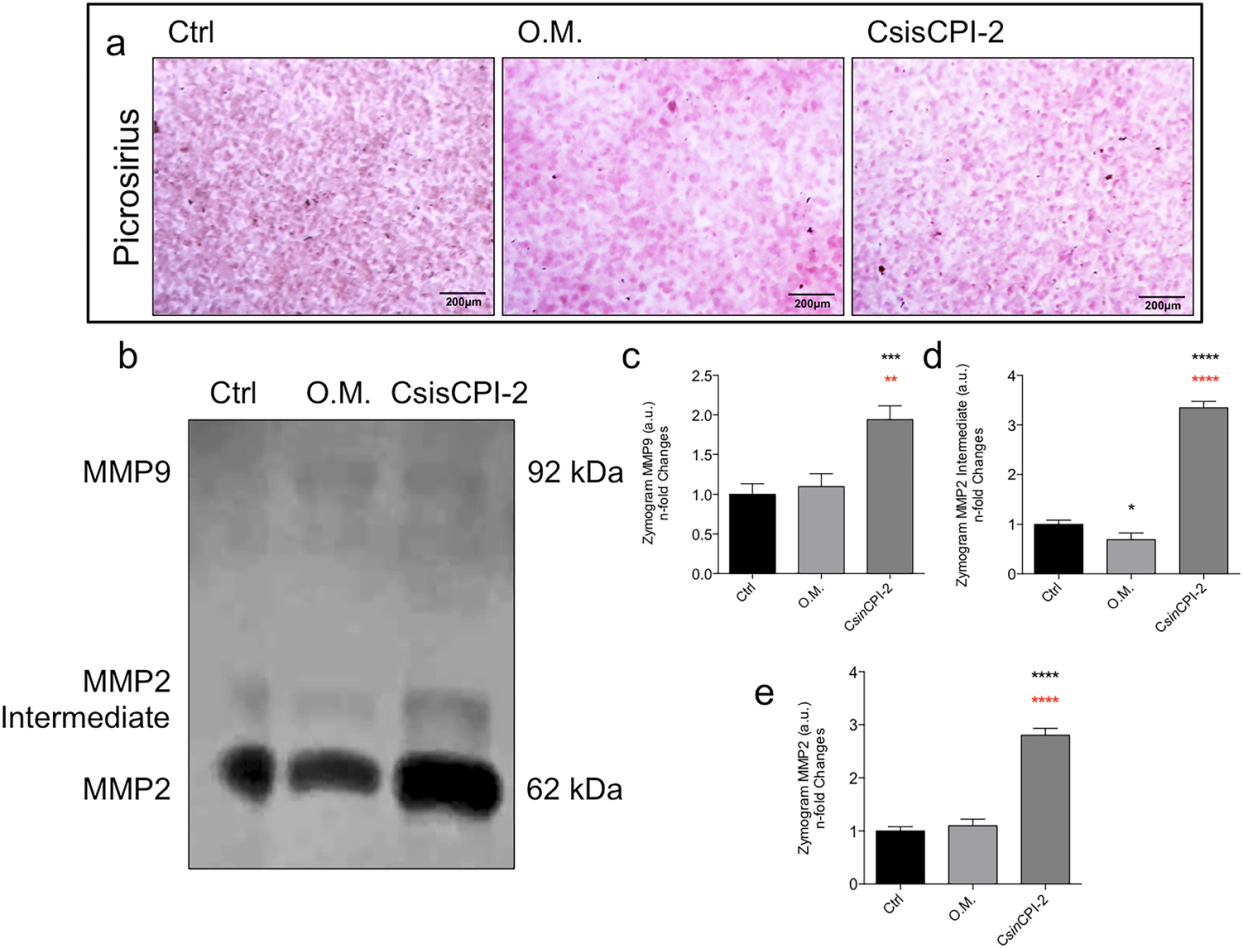
Effect on the remodeling of the extracellular matrix of pre-osteoblasts. After 7 days in the experimental conditions, the cells were marked by Picrossirius (**a**). The culture medium was collected and metalloproteinases (MMPs) evaluated by Zimography gel methodology containing gelatin (**b**–**e**). Statistical difference when compared to the control group: ***p < 0.0002 and ****p < 0.0001. Statistical difference when compared to the Osteogenic Medium (O.M.): **p < 0.0082 and ****p < 0.0001

Considering the non-cytotoxicity of the C*sin*CPI-2 protein and its effect on the pre-osteoblast adhesion process, this prompted us to better investigate whether modulation could be occurring in the differentiation process of the cells used in this study, as well as modulating ECM remodeling. Importantly, activities of MMPs were evaluated by Zymography, where C*sin*CPI-2 promotes a significant increase in the activities of MMP2 and MMP9 when compared to the other experimental groups (Fig. 6b–e).

**Fig. 7:**
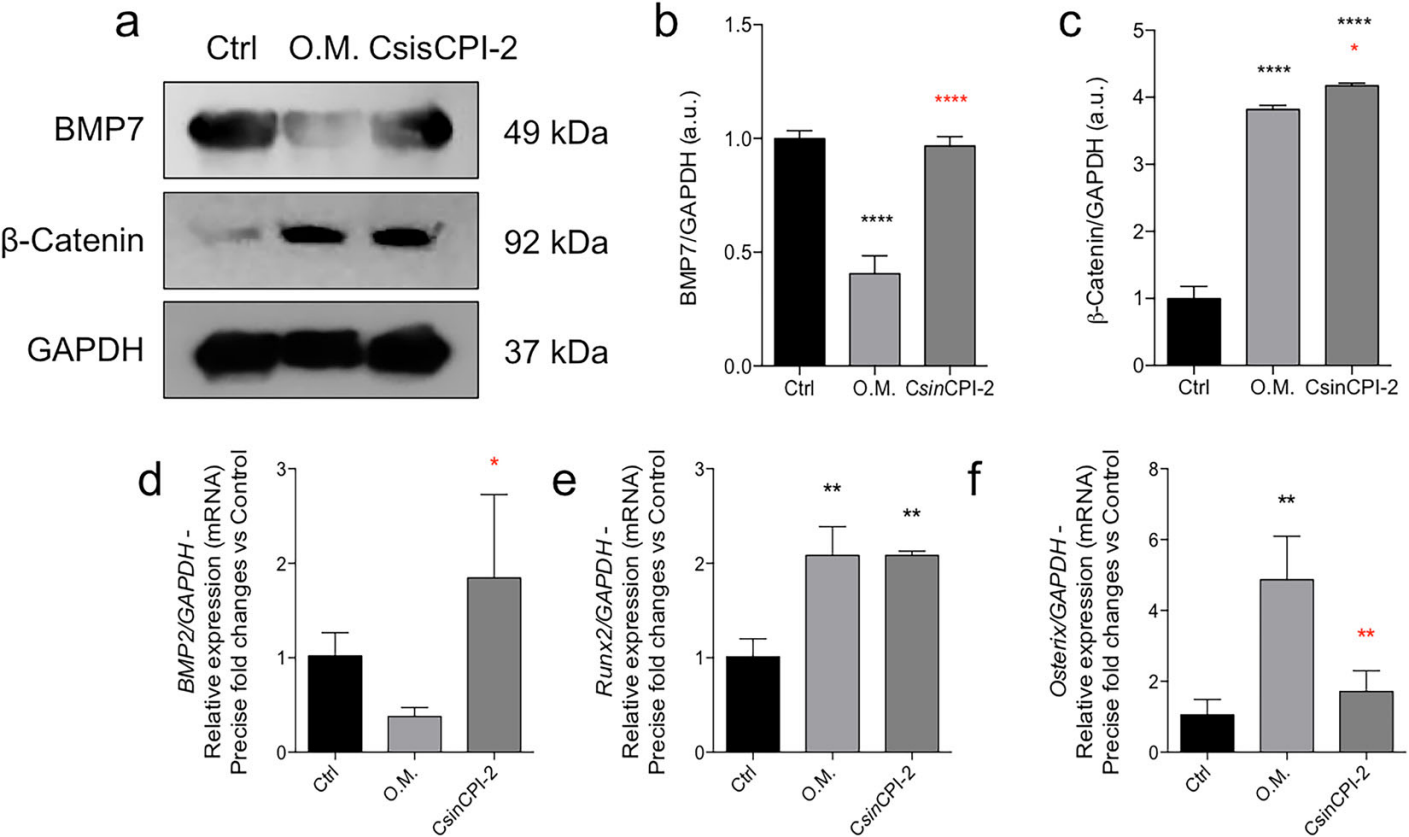
Effect of C*sin*CPI-2 on osteogenic gene biomarkers. The cells were subjected to C*sin*CPI-2 up to 7 days, when the samples were collected to allow protein and mRNA analysis by immunoblotting and qPCR respectively. ß-Catenin and GAPDH proteins were investigated by western blotting (**a**–**c**), while BMP2 (**d**), Runx2 (**e**) and Osterix (**f**) genes were investigated by qPCR technology. GADPH was considered housekeeping gene. Statistical difference when compared to the control group: **p < 0.0082 and ****p < 0.0001. Statistical difference when compared to the Osteogenic Medium (O.M.): *p < 0.05; **p < 0.0082 and ****p < 0.0001

**Fig. 8:**
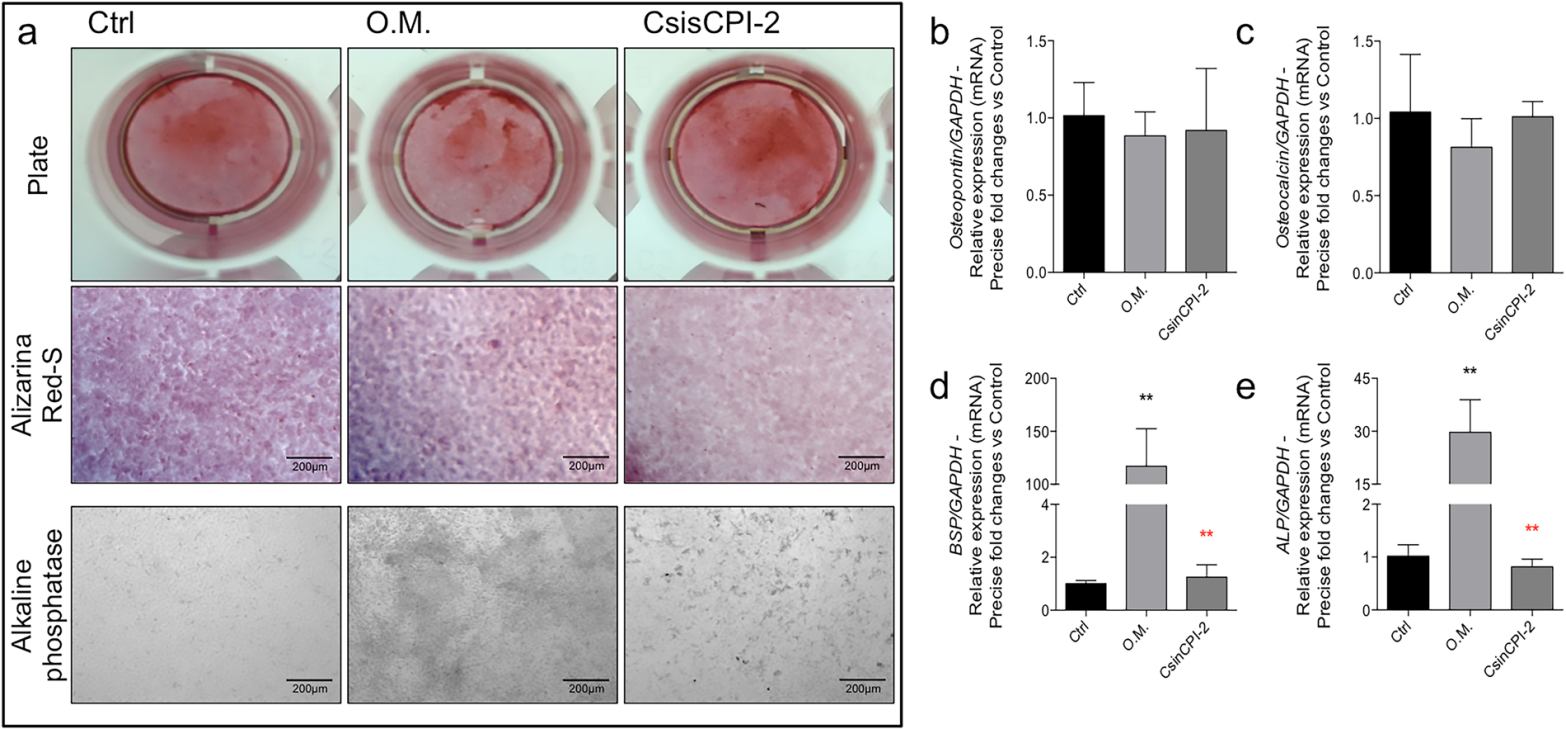
Panorama of genes related with mineralization in vitro in response to C*sin*CPI-2. To assess the mineralization process, the cells were fixed and stained with Alizarin-RedS and Alkaline Phosphatase (**a**). In parallel, the cells were also collected in TriZOL to evaluate the expression of Osteopontin (**b**), Osteocalcin (**c**), BSP (**d**) and ALP (**e**). O.M.: considered a positive control. Statistical difference when compared to the control group: **p < 0.0082. Statistical difference when compared to the Osteogenic Medium (O.M.): **p < 0.0082

Mechanistically, we saw that the protein content of BMP7 was higher than the positive control (osteogenic medium – O.M.), but without statistical difference compared to it ß-Catenin was increased in the groups O.M. in relation to the control and C*sin*CPI-2 in relation to the any other experimental groups (Fig. 7a–c). Still evaluating the differentiation profile, we investigated the gene expression profile of important genes involved with the osteoblastic phenotype, and our data shows there is an increase of BMP2 in response to C*sin*CPI-2 and an increase in Runx2 in the O.M groups and C*sin*CPI2, while Osterix was increased only in the O.M. positive control (Fig. 7d–f), as expected. Genes that regulate calcium metabolism in cell were also investigated, and we found increased expression of both BSP and ALP genes only in the O.M, considered the positive control in the present study. (Fig. 8d–e).

## Discussion

There is a growing trend in the world that seeks minimizing the use of animals as experimental models. In this sense, our group has sought to understand molecular mechanisms, triggered by the external environment of the cell, which aims to better decipher the behavior of bone cells. To that end, we prioritize in vitro testing.

Despite its static appearance, bone tissue has an important and harmful dynamism. The tissue depends on the activity coupled to osteoblasts and osteoclasts, in addition to the paracrine action of other tissues such as, tissues that irrigate and feed surrounding tissue [24–26]. The extracellular matrix (ECM) remodeling processes give the tissue homeostasis of salts and inorganic components [5]. This process affects not only the bone, but other tissues, and when there is no synergistic way it can be affected by the tissue with pathologies [27]. In this sense, cystatin [18, 28] act as key molecules in the process of tissue remodeling contributing with mesenchymal undifferentiated cells that differentiate into osteoblasts [6, 18, 25]. Thus, to better understand this mechanism, pre-osteoblasts were subjected to C*sin*CPI-2 at acute and chronic conditions.

Our findings show that in the first hours of adherence, there is an increase in the expression of genes involved with the cell adhesion process (Integrin-β, FAK and Src) and that it leads to increased expression of Cofilin [29–32]. In addition, we saw a decrease in the content of Phospho-Cofilin (Ser03), a result that suggests an increase in the cytoskeleton remodeling, since p-Cofilin acts by inhibiting the remodeling of the actin filaments [32]. The same genes involved in adherent cell step decreased their expression levels in 24 h, but we saw, in contrast, that kinase-dependent cyclins, CDK2 and CDK4 were increased in the same treatment period. CDK2 is an important gene that promotes the transition of the cell cycle from the Gi to S. CDK4, a member of the IKK4 family was also increased, confirming the transition of the cell cycle between the G1 and S phases [33, 34]. Observing the cell cycle phases it was identified the MAPK-ERK content by western blotting. In 24 h, there is an increase in the Phospho-ERK/ERK ratio, a process that activates kinase activity. It is worth mentioning that ERK participates in several cellular processes, within which we point to a possible proliferative process, indicated by the continuous increase in the content of Phospho-ERK (Thr202/Tyr204) after 48 h of treatment [35, 36]. In addition, ERK may be involved with a cell differentiation process [33], which led us to consider the importance of this process, since studies point to a possible mechanism of action of cystatins as an osteogenic effect [18, 28].

Considering our experimental design, we saw a significant increase in the protein content of β-Catenin. When this protein is in its total form (unphosphorylated), it can be translocated to the nucleus where it acts as a transcription factor for important genes in the osteoblastic differentiation process [37, 38]. We saw that the increase in β-Catenin increased the expression of Runx2 and, at the same time, the expression of Osterix was similar to that found in the control, suggesting a feedback mechanism between the two differentiation markers that increase at different times in the formation of osteoblasts [39–41]. This process is probably being modulated by the action of C*sin*CPI-2. Another important marker of differentiation evaluated here was BMP2 [42]. Some authors show that calvaria-obtained marrow cells from rats treated with cystatin C showed high levels of BMP2 expression when compared to the control group, an action that can positively modulate Runx2 expression [28].

Finally, we saw that at the time determined as cell differentiation, treatment with C*sin*CPI-2 promoted an increase in the activity of Metalloproteinases 2 and 9 (MMP2 and MMP9). Consequently, the protein C*sin*CPI-2 may be acting in the remodeling process of the bone matrix so the mineralization process takes place. This process make feasible collagen fibers in which matrix vesicles can accumulate and deposit calcium, that when complexed with available inorganic phosphate (Pi), forms the essential inorganic complex to mineralize the tissue [5, 43–45].

## Conclusion

Together our data show that in the first hours of treatment, protein in C*sin*CPI-2 promotes an increase in the expression of adhesion markers, which decrease after 24 h, leading to the activation of Kinase-dependent cyclines (CDKs) modulating the transition from G1 to S phases cell cycle. In addition, we saw that the increase in ERK may be associated with activation of the differentiation profile, also observed with an increase in the β-Catenin pathway and an increase in the expression of Runx2 in the group that received the treatment with C*sin*CPI-2. Our results showed an important cascading processes for the formation of bone tissue. The findings corroborate data already listed in the literature and bring a new set of information that opens up an important path for understanding the action of C*sin*CPI-2 on behavior and metabolism of pre-osteoblasts.
